# Understanding and Navigating the Scholarly Communication Landscape in the Twenty-First Century

**DOI:** 10.3389/frma.2019.00004

**Published:** 2019-11-13

**Authors:** Dietmar Wolfram

**Affiliations:** School of Information Studies, University of Wisconsin-Milwaukee, Milwaukee, WI, United States

**Keywords:** research ethics, scholarly communication, big data, research collaboration, open science, research assessment

What is scholarly communication? We lack a universally accepted definition. The Association for College and Research Libraries (ACRL), however, provides a broad perspective:

“Scholarly communication is the system through which research and other scholarly writings are created, evaluated for quality, disseminated to the scholarly community, and preserved for future use. The system includes both formal means of communication, such as publication in peer-reviewed journals, and informal channels, such as electronic mailing lists” (ACRL Scholarly Communications Committee, 2003).

There is no doubt that scholarly communication is a fundamental activity in all scholarly disciplines. Without this system there would be no networks of scholars, dissemination outlets, and the wealth of recorded scholarship would not be available to readers. Simply put, today's knowledge could not advance without scholarly communication. Despite the long history of formal methods for communication, research on how scholars communicate, and the different facets of this system, are needed more than ever. We have entered an exciting and tumultuous time where the research community is grappling with issues that span social and technological concerns. These concerns, in turn, affect the processes and products of scholarly communication that impact the publishers of scholarly works and distributors such as libraries.

Formal processes for scholarly communication have evolved over the centuries, aided by technological developments, and shaped by ideological shifts. Industries and associations have developed around facilitating scholarly communication whether through journals, conferences and associated proceedings, or monographic treatments of research topics. Technologies and new mindsets are transforming—and challenging—traditional systems for the publication and dissemination of scholarly products. One cannot overstate the importance of information and communication technologies (ICTs) for scholars in reducing the barriers of distance and time. Throughout history, the exchange of ideas could only take place as quickly as the fastest mode of delivery. ICTs, ultimately, have made this communication essentially instantaneous, where distance is no longer an obstacle to the sharing of ideas or facilitation of collaborations among scholars across multiple countries.

Similarly, the technologies used to record scholarly discourse, and to collect and store the evidence used in support of research, make it possible to pursue topics on a scale that could only be imagined even a few decades ago. Big data and big science projects now require the efforts of large interdisciplinary teams of researchers who work toward a common goal as part of centralized or decentralized teams. The resources needed to support large projects present their own challenges for researchers, the institutions in which they operate, and the funding agencies that support their work.

Despite the key role of technologies for the storage and dissemination of scholarly products, the system of scholarship is still is most importantly about human-centered activities. Technologies simply provide the tools to facilitate storage, communication, dissemination, and collaboration. The process of scholarship, in turn, is shaped by the environment in which it exists and is influenced by associated cultural norms and ethics of the scholarly community.

How we communicate with colleagues within our disciplines and in other disciplines, and increasingly how what is learned is translated to the public, are more important than ever for accountability and transparency. The Open Science movement encourages all aspects of scholarly activity to be open (de la Fuente, 2018). This movement has been most evident through Open Access publications, where authors or publication venues make the products of scholarship freely accessible to a global audience. Open Science is expanding further to include open datasets, researcher notes, open software, and open peer assessment of scholarly products. Openness encourages accessibility, accountability, reuse, reproducibility, and transparency. This, then, encourages further discovery and understanding. However, the push toward openness can be a double-edged sword. In some disciplinary areas, particularly involving social research, the move for openness must be balanced with the equally important considerations of privacy and confidentiality of personal and social data. This concern extends to big data, where opportunities to discover knowledge can also raise ethical concerns.

Ethical issues are at the forefront of scholarly communication and affect all aspects of scholarly processes. As scholars we strive to reveal and understand the world around us. Increasing pressures for researchers to “produce” have promoted an environment of “publish or perish,” which creates the potential for research misconduct or ethically questionable behavior. The outcomes of the pressures to produce manifest themselves in many forms and may include: the reporting of slipshod research, a focus on the least publishable unit, gratuitous authorship, data fabrication, plagiarism, and selective reporting of results. In scholarly environments where prestige and impact are determined, at least in part, by measures based on citations and other usage data, these pressures extend to journal editors and publishers where increasing the profile of a journal to attract the best submissions presents its own challenges. These issues also extend to peer review, a cornerstone of scholarly communication. With the growing numbers of venues that publish reviewed research, demands on scholars' time to participate in rigorous peer review are increasing (Kovanis et al., 2016).

How we assess scholars and scholarship represents another important facet of scholarly communication in need of further study. Scholarly reward systems encompass both the recognition (or credit) that scholars receive for their contributions as well as the tangible and intangible rewards bestowed upon them that can help advance their careers and stature in the research community. In addition, understanding how scholars remain current and how they engage in information seeking behavior is vital to effective scholarly communication. The growth in the amount of scholarly literature published annually makes it increasingly difficult for researchers to keep up with developments in their own fields, let alone allied disciplines.

With these topics in mind, the Scholarly Communication section of *Frontiers in Research Analytics and Metrics* provides a forum for all aspects of scholarly communication—past, present, and future—that address the stakeholders, processes, products, and the environments in which they exist. We welcome original research submissions that investigate how the changing landscape of scholarship is created and communicated in the sciences, social sciences and humanities, and we encourage interdisciplinary and multidisciplinary perspectives that focus on any of these issues.

## Author Contributions

The author confirms being the sole contributor of this work and has approved it for publication.

### Conflict of Interest

The author declares that the research was conducted in the absence of any commercial or financial relationships that could be construed as a potential conflict of interest.
